# Addressing challenges in routine health data reporting in Burkina Faso through Bayesian spatiotemporal prediction of weekly clinical malaria incidence

**DOI:** 10.1038/s41598-020-73601-3

**Published:** 2020-10-06

**Authors:** Toussaint Rouamba, Sekou Samadoulougou, Fati Kirakoya-Samadoulougou

**Affiliations:** 1 Clinical Research Unit of Nanoro, Institute for Research in Health Sciences, National Center for Scientific and Technological Research, 42, Avenue Kumda-Yoore, BP 218 Ouagadougou CMS 11, Ouagadougou, Burkina Faso; 2 Centre de Recherche en Epidémiologie, Biostatistique et Recherche Clinique, Ecole de Santé Publique, Université Libre de Bruxelles (ULB), Route de Lennik, 808, Bruxelles, 1070 Brussels, Belgium; 3 Evaluation Platform on Obesity Prevention, Quebec Heart and Lung Institute, Quebec, G1V 4G5 Canada; 4 Centre for Research on Planning and Development (CRAD), Laval University, Quebec, G1V 0A6 Canada

**Keywords:** Epidemiology, Statistics, Malaria

## Abstract

Sub-Saharan African (SSA) countries’ health systems are often vulnerable to unplanned situations that can hinder their effectiveness in terms of data completeness and disease control. For instance, in Burkina Faso following a workers' strike, comprehensive data on several diseases were unavailable for a long period in 2019. Weather, seasonal-malaria-chemoprevention (SMC), free healthcare, and other contextual data, which are purported to influence malarial disease, provide opportunities to fit models to describe the clinical malaria data and predict the disease spread. Bayesian spatiotemporal modeling was applied to weekly malaria surveillance data from Burkina Faso (2011–2018) while considering the effects of weather, health programs and contextual factors. Then, a prediction was used to deal with weekly missing data for the entire year of 2019, and SMC and free healthcare effects were quantified. Our proposed model accurately predicted weekly clinical malaria incidence (correlation coefficient, r = 0.90). The distribution of clinical malaria incidence was heterogeneous across the country. Overall, national predicted clinical malaria incidence in 2019 (605 per 1000 [95% CrI: 360–990]) increased by 24.7% compared with the year 2015. SMC and the interaction between free healthcare and health facility attendance were associated with a reduction in clinical malaria incidence. Our modeling approach could be a useful tool for strengthening health systems’ resilience by addressing data completeness and could support SSA countries in developing appropriate targets and indicators to facilitate the subnational control effort.

## Introduction

Since the 2000s, with the support of technical and financial partners, Burkina Faso has undertaken numerous initiatives and reforms to improve population health. These various initiatives and reforms have been mainly implemented at the operational level of the health system (health district) and include both the strengthening of health system capacities and disease-specific control programs^1–5^. To continuously build and sustain good management of the health system, it is essential to obtain estimates from the analysis and interpretation of quality routine data for rational planning, appropriate resource allocation, evidence-based policy making, effective monitoring of health service delivery and policy evaluation^6–9^. Currently, in Burkina Faso, the routine health data collection tools in the framework of the health management and information system include both the Official Weekly Telegram (OWT) and the National Health Data Warehouse (ENDOS-BF), which is derived from District Health Information Software 2 (DHIS2)^1,10^. The OWT is the national epidemiological surveillance system tool for early warning, forecasting and response to disease outbreaks, whereas the ENDOS-BF focuses on the health facilities’ monthly activity reports and aims to facilitate the management of health resources and editions of yearly health statistics books.

With the malaria data from the OWT (at least 5 years of weekly data to define the expected “long-term” weekly caseload), several approaches were proposed by the WHO to calculate thresholds for early detection of malaria epidemics. Four methods of calculation are mainly used: constant case count, mean + two standard deviations (2 SD), median + upper third quartile and cumulative sum^8^. In the Burkina Faso setting, the main use of malaria data from the OWT is the production of weekly malaria surveillance bulletins, which include a comparison of the absolute count of malaria cases and deaths per week relative to the weeks of the previous three years^3,10,11^.

To translate routine health data into information to support the design of health interventions, it is essential to establish a surveillance system that is resilient, consistent and comprehensive. Unfortunately, health systems in most sub-Saharan African (SSA) countries, in addition to their structural and resource challenges, are often vulnerable to unplanned situations that cripple effectiveness in managing disease outbreaks and control. Socio-political troubles may acutely affect routine data reporting from health facilities, resulting in significant gaps in national health data^2,12^. Indeed, in Burkina Faso in 2019, the reporting of routine health data was deliberately interrupted for half the year due to an unusually long strike of healthcare workers. This was especially detrimental to the malaria control program since the strike spanned the transmission period. In such conditions, to address missing or non-reported surveillance data, multiple imputation methods (widely used for handling missing data in biomedical research) can be used if relatively few data points are missing from the time series. However, when the data collection and reporting are interrupted for a longer time interval, or when multiple variables have missing values, imputation methods are limited because these classical approaches do not take into consideration the temporal and spatial structures in the data^13–15^.

In Burkina Faso, the prediction of health data, including malarial indicators at the operational level, has usually been performed on the basis of ordinary least squares (OLS)^16^. The routine surveillance data have specific features that limit the optimal use of this classical statistical method^17^. Statistical models with spatial and temporal correlation structures implemented in a Bayesian framework have been shown to better fit these types of data. This approach can take into account missing data, weighting estimates according to observed or unobserved covariates that are close in space and time, and allows the inclusion of suitable covariates while accounting for uncertainty^17–20^. Although Bayesian spatiotemporal methods can generate reliable and accurate estimates of the malaria burden and measure progress in malaria control, their implementation and interpretation require a certain level of statistical skills, and these skills are not always present in malaria control programs^21,22^.

The World Health Organization (WHO) recommends transforming malaria surveillance as core information (pillar 3) to support the control and elimination of malaria^8,9,23^. Surveillance data are mainly influenced by factors related to population access to healthcare, care-seeking behavior, health facility readiness to manage and report malaria cases and remote sensing weather data. These different sources of data could be coupled to appropriately estimate clinical malaria incidence, assess progress made in malaria control through modeling, and predict the disease spread^24–27^.

In addition, these models could provide valuable support to promptly pinpoint the areas where the malaria burden is high (in terms of an excess of risk) and assess the effect of health programs on spatial and temporal trends^28,29^. To the best of our knowledge, few studies have investigated the prediction or forecasting of clinical malaria incidence at a subnational level in an aim to address the completeness of routine health data reporting. Previous studies on the subject have often used statistical methods such as generalized linear models, auto-regressive integrated average models, or Holt–Winters models^27,30–37^.

To fill this gap, we propose an analysis approach to estimate weekly clinical malaria incidence for periods during which routine data were not collected. To this end, this study aimed to link weekly malaria data and weather, as well as health programs (seasonal malaria chemoprevention and free healthcare) and contextual predictors, and then apply Bayesian spatiotemporal modeling to predict weekly clinical malaria incidence in each health district for the year 2019. In the present study, we assumed that malaria transmission during the health workers strike would be similar to that experienced in previous years.

## Methods

### Setting

This study involves all the health districts of Burkina Faso. Burkina Faso is an SSA landlocked country located in the heart of West Africa and shares borders with six countries. It lies between 9°20′ and 15° north latitude and the longitudes 2°30′ east and 5°30′ west with a surface area of 272,960 km^2^. Only a small proportion of the territory is inhabited, with a high density of the population in the two largest cities, Ouagadougou and Bobo-Dioulasso. The climate of Burkina alternates between a dry season from November to June and a rainy season from July to October. The country is classified among the poorest countries in the world. The epidemiological profile is characterized by endemic infectious diseases (occasionally becoming epidemic) with a progressive increase in the burden of non-communicable diseases. All health facilities in the country (public [number increased from 1897 to 2286 in 2011 and 2019, respectively] and private [number increased from 381 to 533 in 2011 and 2019, respectively]) are grouped into health districts. In 2015, the number of health districts in the country increased from 63 to 70^1,38^. The health district unit represents the operational entity of the national health system and is the level at which several health programs were implemented and coordinated. Healthcare is provided by public and private structures. Malaria has remained the leading cause of medical consultation and death in medical centers and hospitals since 2010^38^. Several recent specific health programs and nonspecific health programs were implemented at different time points and paces to reduce the burden of malaria. The target was to reduce malaria case incidence by at least 40% nationally in 2020 compared with 2015^3,9^. These programs mainly included seasonal-malaria-chemoprevention (SMC) among young children, intermittent preventive treatment during pregnancy (IPTp), free healthcare for young children and pregnant women, campaigns for the distribution of long-lasting insecticide-treated nets (LLINs) and home-based management of malaria by community-based health workers^39^.

### Data sources and data description

In this study, we used health district weekly malaria surveillance data and several other data sources aggregated by health district, namely, weather and health program data, as well as health district contextual factors.

#### Health district clinical malaria cases

The Ministry of Health of Burkina Faso, through the National Malaria Control Program (NMCP), provided the malaria data used in this study. The current malaria data reported in the OWT are a combination of parasitological confirmed and clinically diagnosed cases. It is noticeable that before July 2018, this database included all malaria cases (uncomplicated and severe cases) and death cases reported by both public and private health facilities regardless of gender and age. In July 2018, with the coverage of SMC in 65 health districts of the country, the weekly malaria data were stratified to have specific data for children under 5 years of age. This was done with the aim of assessing the effect of SMC on the targeted population^40^. For our analysis purposes, we considered all data on weekly malaria cases (ranging from uncomplicated to severe cases) recorded in Burkina Faso OWT between January 2011 and May 2019.

#### Control variables

To account for control variables, we followed previous studies to model the relationship between malaria and its associated factors^20,29,41–43^. Table 1 outlines the factors that might influence the distribution of malaria, as well as the period for which data on each factor are available and the level of spatial aggregation.

- Health district capital city: daily weather data (rainfall, temperature and relative humidity, all measured at 2 m from ground level) from January 2011 to December 2019 were downloaded from the US NASA (United Stated National Aeronautics and Space Administration) site^44^ and then aggregated by week according to the epidemiological calendar of the Burkinabe Ministry of Health.
- We used data from two new main health programs that substantially influenced the distribution of malaria burden^29,42,43^, namely the SMC strategy and the free healthcare policy. Since the SMC strategy was progressively implemented from 2014 (in 7 health-districts) to 2018 (in 65 districts) before reaching full coverage in 2019, we accounted for the duration that this strategy was in place in each health district. For the free healthcare, we used this information as a binary variable: the period before free healthcare implementation (before May 2016) and the period after the implementation (after May 2016). However, it is notable that before the scaling up of free healthcare, pilot experiments on free healthcare financed by several partners were already running in a limited number of health districts (Tougan, Séguénéga, Dori, Sebba, Diapaga, Fada, Kaya)^45^.
- The availability of rapid malaria tests (RDTs) in Burkina Faso health districts was estimated from periodic national surveys on the performance of health facilities (SARA) carried out in 2012, 2014 and 2016. The SARA data were obtained freely from the Ministry of Health of Burkina Faso. Since the sample sizes for these surveys were representative of the regional administrative boundaries, the estimates at the health district level were obtained through binomial models implemented in a Bayesian framework^29,46,47^. It should be noted that according to the national policy, the diagnosis of malaria at first-level health centers is based mainly on RDTs (sometimes by clinical symptoms if RDTs are unavailable), and diagnosis is often made by microscopy in reference health centers with a laboratory equipped with this apparatus, i.e., medical centers, district hospitals, regional hospitals and university hospital centers.
- In our study, the whole population within each health district was considered to be at risk. The number of inhabitants in Burkina Faso per health district and per 100 m^2^ grid were also extracted from worldPop^48^. The entire population of each health district was obtained through the sum of the value per 100 m^2^ according to the area of each health district.
- The proportion of children under five years of age and pregnant women: since pregnant women and children under five years of age constitute the most vulnerable populations to malaria, the modeling considered these subpopulations. The number of pregnant women per 1 km^2^ grid and the number of children under five years of age per 100 m^2^ grid in each health district between 2011 and 2019 were extracted from worldPop^49^. These indicators were included in the model as adjustment variables by considering the percentage of these estimated high-risk groups relative to the whole population in each health district.
- We also used the number of new contacts at the health facilities per capita per year. In this study, the term “new contacts” included all people attending health facilities for any cause of disease, including pregnant women attending their antenatal visits. These annual data were extracted for each health district from the national health statistical yearbooks edited by the Ministry of Health. With this indicator, a proxy of the health facility attendance rate was computed by dividing the annual number of medical consultations over the number of the total population in each health district^38^.
- The data on poverty (proportion of households in the lowest wealth quintile) were obtained from the results of the demographic and health survey (DHS) conducted in 2010, 2014 and 2017–2018. Since DHSs are designed to generate estimates at the national and regional levels, data on the proportion of the poorest households were estimated at the district level through binomial models implemented in a Bayesian framework^29^.
- The distance (in kilometers) to the nearest inland waterbody within each health district was obtained from WorldPop^50^ at a spatial resolution of 100 m^2^. The shortest path (geodesic distance) from each grid cell center to the nearest inland waterbody was computed through the haversine formula. For this distance estimation, a global gridded inland waterbody dataset was used to prevent edge effects at the country boundaries^50^.

**Table 1:** Factors purported to influence the spatiotemporal dynamics of malaria.

Factors	Period	Temporal unit	Original Scale	Data source
**Weather factors**				
Temperature^†^ (min average, max) in °C	2011–2019	Weekly	District	NASA^44^
Average rainfall^†^ (millimeters)	2011–2019	Weekly	District	NASA^44^
Average number of rainfalls event (count)	2011–2019	Weekly	District	NASA^44^
Average relative humidity^†^ (%)	2011–2019	Weekly	District	NASA^44^
**Health program factors**				
Free-of-charge of healthcare (before vs. after)	2016–2019	Annual	District	MoH
Duration (in years) of SMC program	2014–2019	Annual	District	MoH
**Health district-specific factors**				
Availability of malaria rapid diagnosis test	2012–2016	Biannually	Regional^¥^	MoH
Number of total inhabitants	2011–2019	Annual	100 m^2‡^	WorldPop^48^
Number of children under five years of age	2011–2019	Annual	100 m^2‡^	WorldPop^48^
Number of pregnant women	2011–2019	Annual	1 km^2‡^	WorldPop^48^
Health facility attendance rate	2011–2019	Annual	District	MoH
Proportion of households in the lowest wealth quintile	2010–2018	Quadrennial	Regional^¥^	DHS program
Distance to the nearest inland waterbody	2015	–	100 m^2‡‡^	WorldPop^48^

*SMC* seasonal chemoprophylaxis, *NASA* National Aeronautics and Space Administration (US).

^**†**^Measured at 2 m from the ground level (surface air).

^¥^The original scale was regional, the estimates at the health district level for our study purposes were obtained through binomial models implemented in a Bayesian framework.

^‡^The values for the entire health district were obtained through the sum of the values per 100 m^2^ or 1 km^2^ according to the area of each health district.

^‡^The values for the entire health district were obtained through the arithmetic mean of the value per 100 m^2^ according to the area of each health district.

### Statistical analysis

#### Assessing temporal and spatial autocorrelation in the crude data of malaria

The crude clinical malaria incidences at health district level $$k$$ at a given time $$t$$ were calculated by dividing the number of malaria cases $$Y$$ by the total size of the population at risk, $$P$$$${Incidence}_{kt}= {Y}_{kt}/{P}_{kt}$$, where $${Y}_{kt}$$ is the observed number of clinical malaria cases and $${P}_{kt}$$ is the population at risk in health district *k* at week *t*.

Temporal autocorrelation: we detected temporal autocorrelation by looking at the autocorrelation plots of the residuals of the model from the seasonal autoregressive integrated mobile average (SARIMA), using a non-spatial Poisson and a non-spatial over-dispersion model.

Spatial autocorrelation: we fitted a model that included all predictor variables while ignoring spatial correlation and then analyzed the structure of residuals.

#### Bayesian spatiotemporal modeling

##### Null model fit

After assessing several exponential family distributions, we retained a negative binomial space–time model to estimate the weekly clinical malaria incidence rate at the health district level.

A negative binomial Hierarchical Bayesian spatiotemporal model with the log link function was designed to include weather, health programs and contextual predictors. Spatial random effects (${\psi }_{k}$) and temporal random effects (${\upomega }_{t}),$ as well as their interactions (${\delta }_{kt}$), were included in the model.

Thus, the number of malaria cases ${Y}_{kt}$ observed in district $k$ = 1,…,70 at week $t$ = 1,…,418,…,469 follows a negative binomial distribution such that:

1 $${Y}_{kt}\sim Negative \, Binomial ({\pi }_{kt}, \vartheta )$$

2 $${\pi }_{kt}= \vartheta / (\vartheta +{\mu }_{kt})$$

where $\vartheta$ is the dispersion parameter and ${\mu }_{kt}$ is the mean weekly number of malaria cases in health district *k*.

The specification of the model structure was determined by testing a series of different combinations of temporal (AR1, AR2, RW1, RW2) and spatial (Besag, Besag–York–Mollié) random effects and then using the most parsimonious model based on the value of the lowest deviance information criterion (DIC). We analyzed ${Y}_{kt}$ using the Besag–York–Mollié (BYM) method to model the spatial random effects^51^. BYM considered both structured *(*${u}_{k}$) and unstructured (${v}_{k}$) spatial random effects in the modeling. Similarly, we modeled the temporal random effects (secular trend from 1 to 469 weeks) by using an autoregressive process order 2 (AR2) to capture the structured temporal random effects (${\gamma }_{t}$), whereas the unstructured temporal random effects were modeled via Gaussian distribution. From the spatial and temporal structure, the space–time interaction (${\delta }_{kt}$) between the two unstructured components (${{v}_{k}\;\mathrm { and }\;\gamma }_{t}$) was included in the model. Finally, we included an annual trend (${\rm T}_{j}$) and annual seasonal (${S}_{j}$) random effects for each year $j$ (j = 1,…8, 9) to account for inter-annual variance and annual seasonality not directly captured by other components of the model. The underlying malaria risk ${\mu }_{kt}$ without covariate effects is modeled as follows:

3 $$log\left({\upmu }_{kt}\right)={\beta }_{0}+\mathit{log}\left({P}_{kt}\right)+{\psi }_{k}+{\upomega }_{t}+ {\delta }_{kt}+{\mathrm{\rm T}}_{j}+{\mathrm{S}}_{j}+ {\varepsilon }_{kt}$$

4 $${\psi }_{k}={u}_{k}{+v}_{t}; \,\,{\omega }_{t}={\gamma }_{t}+{\phi }_{t}$$

where ${P}_{kt}$ is the offset health-district population, ${\beta }_{0}$ is the intercept (i.e., the overall log malaria risk over the study period in Burkina Faso), and ${\varepsilon }_{kt}$ captures supplementary variability in the data not described by other model components.

Prior distributions were assigned for all model parameters. A non-informative prior was assigned to the regression coefficients (${\varvec{\beta}}\sim N(0, 1/{\tau }_{\beta })$). We used the prior conditional autoregressive (CAR) for the structured spatial random effects (${u}_{k}\sim CAR({\tau }_{u})$) with a spatial adjacency matrix W of size 70 × 70 (where ${w}_{kj}$ = 1 if health districts *k* and *j* share a common boundary, and 0 otherwise), whereas a prior Gaussian distribution was used for the unstructured spatial random effects (${v}_{k}\sim N(0, {\tau }_{v})$). Similarly, we used the prior autoregressive for the structured temporal random effects (${\gamma }_{t}|{\gamma }_{t-1}|{\gamma }_{t-2}\sim N({\gamma }_{t-1}+{\gamma }_{t-2}, {\tau }_{\gamma })$) and the prior Gaussian distribution for the unstructured temporal random effects (${\phi }_{t}\sim N(0, {\tau }_{\phi })$). A prior Gaussian distribution (${\delta }_{t}\sim N(0, {\tau }_{\delta })$) was assigned to the space–time interaction term.

The model specification used minimally informative priors on the log of structured and unstructured effect precision, $\mathrm{log}\left({\tau }_{u}\right), {\mathrm{log}(\tau }_{v})\sim logGamma\left(1, 0.0005\right)$. A non-informative Gaussian prior distribution was used for weekly temporal, annual temporal and seasonal random-effects precision, ${\tau }_{\gamma }^{2}, {\tau }_{\phi }^{2}, {\tau }_{\delta }^{2},{\tau }_{S}^{2} {\tau }_{T}^{2}\sim Gaussian\left(1, 0.01\right)$.

##### Inclusion of covariates in the hierarchical Bayesian spatiotemporal model

The null model was adjusted to consider covariate effects (${\varvec{X}}$) on the weekly clinical malaria incidence rate variation. The selection of variables for the construction of the final model was performed based on factors that are purported to influence the distribution of malaria, as reported frequently in previous studies^20,29,41,42^. To handle the problem of multicollinearity existing between the weather variables, we performed a principal component analysis (PCA) and retained the number of components, which explained more than 70% of the total inertia. (Figure S1). In addition, we centered and reduced (normalizing to a mean of 0 and standard deviation of 1) certain continuous variables (proportion of children under five years of age, proportion of pregnant women and distance to the nearest inland waterbody). Then, covariates were included in the model incrementally, and the DIC was checked step by step to determine whether its value had improved. The values of the DIC are given in Table S1. The final underlying malaria risk ${\mu }_{kt}$ with covariate effects was modeled as follows:

5 $$log\left({\upmu }_{kt}\right)={\beta }_{0}+\mathit{log}\left({P}_{kt}\right)+{\psi }_{k}+{\upomega }_{t}+ {\delta }_{kt}+{\mathrm{\rm T}}_{j}+{\mathrm{S}}_{j}+{{\varvec{\beta}}}_{{\varvec{w}}}{{\varvec{X}}}_{{\varvec{k}}{\varvec{t}}}+{{\varvec{\beta}}}_{{\varvec{H}}{\varvec{P}}}{{\varvec{X}}}_{{\varvec{k}}{\varvec{j}}}+{{\varvec{\beta}}}_{{\varvec{e}}{\varvec{n}}{\varvec{v}}}{{\varvec{X}}}_{{\varvec{k}}}+{\varepsilon }_{kt}$$

${\beta }_{{\varvec{w}}}$ is a vector of regression coefficients associated with the vector of weekly weather predictors (${X}_{{\varvec{k}}{\varvec{t}}}$: rainfall, number of rain events, temperature, relative humidity), ${\beta }_{{\varvec{H}}{\varvec{P}}}$ is a vector of regression coefficients associated with the vector of annual health program predictors and contextual variables (${X}_{{\varvec{k}}{\varvec{j}}}$: SMC duration, free health care, poverty index, children under five years of age and pregnant women, health facility attendance rate, malaria rapid diagnosis test), and ${\beta }_{{\varvec{e}}{\varvec{n}}{\varvec{v}}}$ is the vector of regression coefficients associated with the vector of the permanent predictor (${X}_{{\varvec{k}}}$: average distance to land water). We included an interaction between the free healthcare and health facility attendance rate in order to study the main effects along with their interaction effect.

##### Prediction of weekly malaria cases

The major objective was to predict the trend of weekly malaria cases in each health district; this prediction was made as a part of the model fitting itself. Since prediction was the same as fitting a model with some missing data (covering the period from January to December 2019), we set the dependent variable, ${Y}_{kt}$, as missing for those 52 weeks in the health districts of interest with missing data. Then, we introduced the covariate values to the model, as well as the spatial, temporal and seasonal random effects. We also included the space–time interaction covering the time to be predicted (weeks 418–469). The median and the 2.5% and 97.5% quantiles of weekly fitted values were used as the prediction estimates for each health district with a 95% credible interval (95% CrI). Finally, from the end result of weekly predicted values, the annual clinical malaria incidence rate per 1000 inhabitants in 2019 for each health district was estimated. These estimates were compared with the incidence rate in the year 2015 to quantify both the whole nation and health district-specific progression of the clinical malaria incidence trend.

##### Model goodness-of-fit

After taking into account the covariate effects, several measures were used to assess the accuracy of the weekly malaria prediction model. The conditional predictive ordinate (CPO) and Cramer–von Mises test of goodness-of-fit (null hypothesis: "*uniform distribution*") was used to check the internal validation of the models^52,53^. Furthermore, we deleted some data (about 12.5%, corresponding to about 52 weeks) at random from the dependent variable (${Y}_{kt}$) and executed the model with these missing values. Then, we used the resulting predicted values to check whether the model was still accurate in predicting the actual values. Similarly, we validated the ability of our model to predict an entire transmission season for one of the years for which we had complete data. The convergence of the model (covariates) was monitored graphically. We used three metrics to assess the accuracy of the model. First, we calculated Spearman correlation coefficient between the actual observed data collected during the first quarter (20 weeks) of the year 2019 and the predicted values of clinical malaria cases during the same period. Second, we calculated the R^2^ from the ordinary least squared model. Third, we calculated the root-mean-squared error (RMSE).

All data analyses and maps design, were carried out using R software (R Foundation for Statistical Computing, Vienna, Austria).

### Ethics

The study was approved by the National Ethics Committee ("Comité d’Ehtique pour la Recherche en Santé (CERS)") of Burkina Faso (Approval reference: No. 2019-79/MS/MESRSI/CERS du 19 July 2019). All methods mentioned in the study protocol were carried out in accordance with the CERS guidelines and regulations. The data used in this analysis are health facility aggregated data reported in the Official Weekly Telegram and provided by the National Malaria Control Program. The data analysis was carried out at the health district level with no reference to individual-level identification particulars (i.e. individuals are health facilities not human directly).

## Results

### Temporal trends and spatial distribution in the crude incidence of clinical malaria

Table 2 shows the national annual variation in the clinical malaria incidence rate between 2011 and 2018. Overall, there was an increase in reported cases of clinical malaria nationwide. Over the eight years, the cumulative annual average incidence rate of clinical malaria increased from 347 in 2011 to 588 cases per 1000 inhabitants in 2018, which represents an average increase of eight percent per year. Moreover, from 2016 onwards, it was noted that the minimum incidence rate reported by a district became higher than 150 per 1000, in contrast to previous years, during which it was around 20 per 1000.

**Table 2:** Summary of national annual reported malaria cases in Burkina Faso between 2011 and 2018.

Year	Population^a^	Total malaria cases^b^	Annual cumulative clinical malaria incidence rate per 1000 inhabitants					
			Min	Q1	Median	Mean	Q3	Max
2011	16,019,684	5,550,955	18	259	362	347	452	763
2012	16,507,009	6,634,433	11	313	450	402	527	814
2013	17,007,066	6,985,723	30	327	427	411	550	859
2014	17,518,939	8,083,435	27	401	509	461	595	924
2015	18,041,723	8,128,823	28	372	472	450	584	1027
2016	18,575,538	9,766,294	224	458	552	526	640	946
2017	19,121,074	11,405,735	241	515	631	597	763	1202
2018	19,677,916	11,576,369	161	532	633	588	781	1117

^a^Source: worldPop^48^.

^b^Malaria cases (mix of confirmed and clinically diagnosed cases) reported in the National Official Weekly Telegram.

The national weekly clinical malaria incidence rate was not uniformly distributed throughout the weeks of the year. The incidence rate peaked annually during the rainy season with a lag time of six to eight weeks from the beginning of the rainy season, i.e., the week of the first rainfall of the rainy season period (Figure S2).

**Figure 1:**
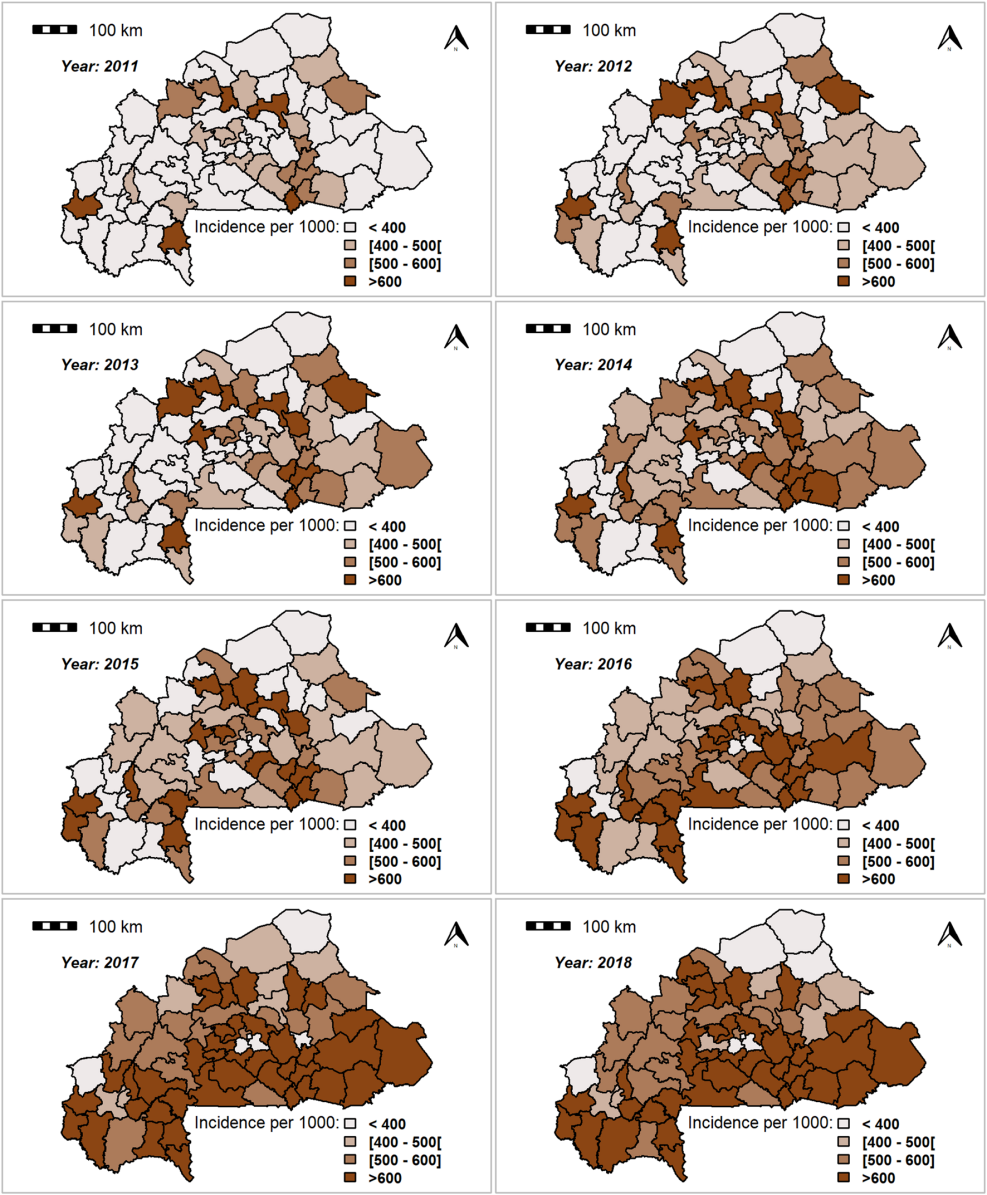
The clinical malaria incidence for each health district of Burkina Faso from 2011 to 2018. Clinical malaria incidence is the number of cases per 1000 inhabitants. The number of malaria cases for each district of Burkina Faso from 2011 to 2018 was collected from the national Official Weekly Telegram. The population data for each district of Burkina Faso were downloaded from worldPop^48^. Maps created by Rouamba T. et al., 2020.

Figure 1 shows the spatial distribution of crude malaria annual incidences in all 70 health districts of Burkina Faso from 2011 to 2018, while the absolute number of cases is presented in the Supplementary Material (Figure S3). The annual crude cumulative incidence rates increased year by year and were higher in health districts located in the south, east and south-west compared with the other health districts in the country. Overall, the annual crude total number of clinical malaria cases over the study period was high in areas with large populations (Ouagadougou, Bobo-Dioulasso, Ouahigouya, Fada N’Gourma), while small numbers of cases mostly occurred in areas with small populations. The crude incidence rates in these health districts with large populations exhibited lower values compared with the other health districts.

### Bayesian model validation and effects of covariates

Table 3 presents the parameters of the models (modelled from Eqs. 3 and 5 above). The DIC of the full model (DIC = 449,843) was 16,266 points lower than that of the null model (DIC = 466,109). The convergence plot of the covariates of the model is presented in Figure S4. After adjusting for weather factors, children under five years of age, pregnant women and distance to the nearest inland waterbody, the SMC program and the interaction effect between free health care and health facility attendance rate appeared to be associated with a decrease in clinical malaria incidence. The findings showed that a health district that had one additional year of the implementation of the SMC program (primary predictor) reduced the clinical malaria incidence by 7.2% (95% CrI 6.6–7.9%). The quantification of the effect of the interaction effect between free healthcare and health facility attendance rate showed that the clinical malaria incidence risk was reduced by 53.6% (95% CrI 52.0–55.1). A significantly higher clinical malaria incidence appears to be associated with a higher rate of availability of malaria rapid diagnosis test (adj. RR = 1.13, 95% CrI 1.09–1.18). Similarly, a higher proportion of households in the lowest wealth quintile was associated with an increase in clinical malaria incidence rate (adj. RR = 1.05, 95% CrI 1.00–1.10).

**Table 3:** Posterior estimates of Bayesian model parameters and covariable effects on malaria.

Parameters	Posterior estimate	
	Null model	Full model
**Bayesian model validation**		
Posterior mean of the deviance ($\stackrel{-}{\mathrm{D}}$)	465,681	444,267
Deviance information criterion (DIC)	466,109	449,843
Effective number of parameters (P_D_)	4289	5567
Spatial fractional variance (%)	99	74
**Precision of random effects of the model (**median, 95% CrI**)**		
${\uptau }_{\mathrm{\vartheta }}$ (parameter for zero-inflation)	2.39 (2.35–2.43)	1.67 (1.66–1.69)
${\uptau }_{\mathrm{u}}$ (spatial structural random effects)	2.95 (2.39–3.45)	1.31 (0.97–1.80)
${\uptau }_{\mathrm{v}}$ (spatial unstructured random effects)	4.30 (3.50–5.27)	7.22 (4.92–8.86)
${\uptau }_{\upgamma }$ (temporal structural random effects)	3.53 (3.28–3.72)	2.04 (1.17–2.69)
${\uptau }_{\upphi }$ (temporal unstructured random effects)	9.43 (7.27–10.87)	9.67 (7.94–11.29)
${\uptau }_{\mathrm{S}}$ (seasonal random effects)	3.87 (3.73–4.03)	9.43 (7.14–11.10)
${\uptau }_{\updelta }$ (space–time random effects)	5.82 (5.51–6.07)	5.89 (5.58–6.14)
${\uptau }_{\mathrm{T}}$(annual temporal random effects)	9.09 (6.40–10.77)	9.59 (7.02–11.18)
**Fixed effect on antilog scale, relative risk (**median, 95% CrI**)**		
Duration (years) of seasonal chemoprevention of malaria		0.93 (0.92–0.94)
Availability of malaria rapid diagnosis test		1.13 (1.09–1.18)
Proportion of households in the lowest wealth quintile		1.05 (1.00–1.10)
Main effects of free-of-charge of health care		
Period before free healthcare		1
Period after free-of-charge of health care		2.27 (2.03–2.54)
Main effects of health facility attendance rate		5.81 (5.67–5.96)
Interaction effect between the presence of free healthcare and facility attendance rate		0.46 (0.45–0.48)

CrI, credible intervals; model estimates are adjusted for rainfall, temperature and relative humidity, population, proportion of children under five years of age, proportion of pregnant women and distance to the nearest inland waterbody.

### Bayesian results for yearly spatiotemporal dynamics of clinical malaria incidence

**Figure 2:**
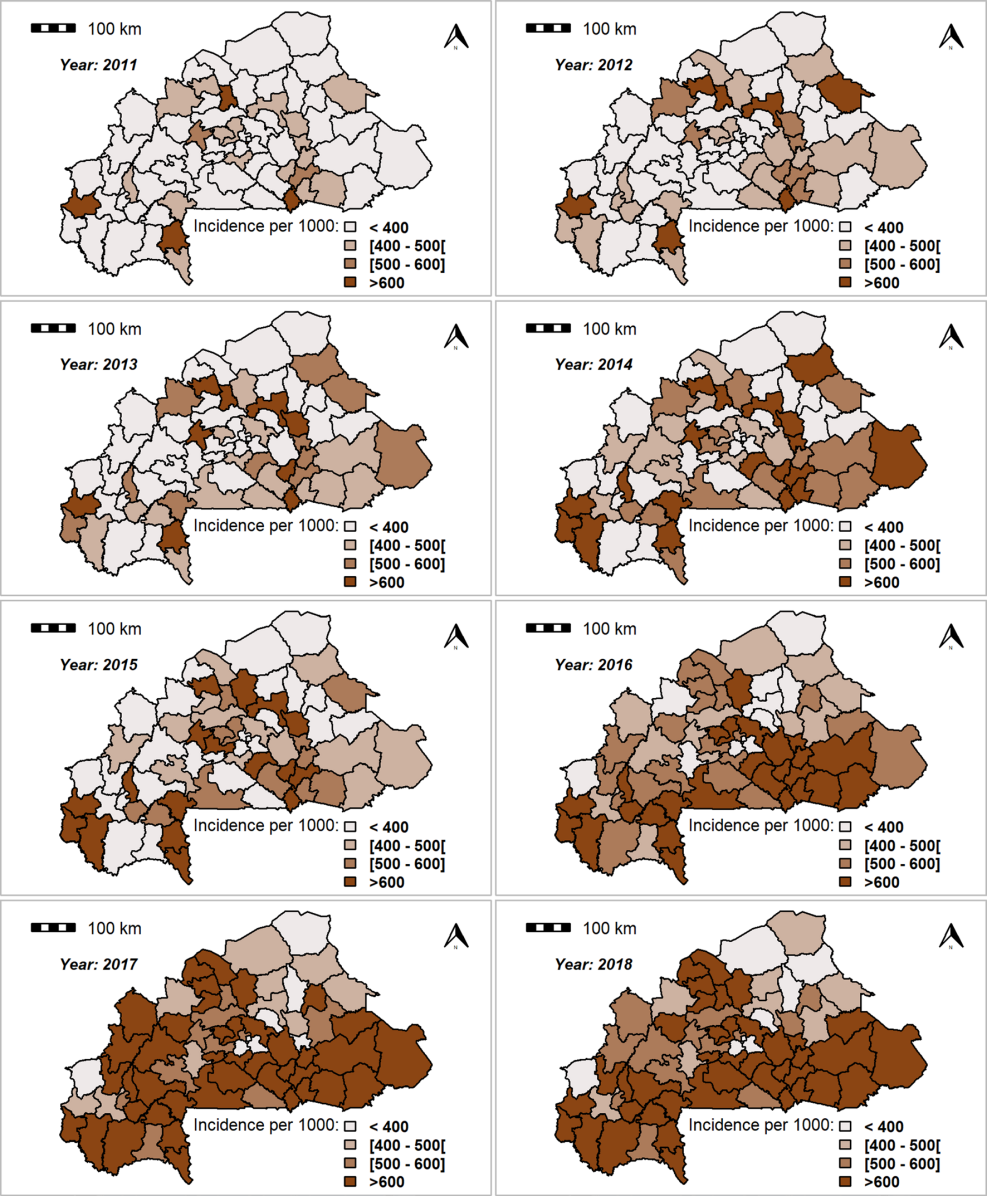
Spatiotemporal dynamics of annual clinical malaria incidence as a rate (per 1000) of fitted values (based on posterior medians) between 2011 and 2018. Source: the shapefile was obtained from the “Base Nationale de Découpage du territoire” of Burkina Faso (BNDT, 2006). Maps created by Toussaint Rouamba et al., 2019.

The results show that there was a strong spatial dependence in clinical malaria incidence, as suggested by the spatial fractional variance value presented in Table 3. The fitted incidence rates increased and followed the same trends compared with the crude incidence. It is noticeable that the incidence increased year by year and was higher in health districts located in the south, east and south-west parts of the country (Fig. 2).

### Accuracy of the clinical malaria case prediction

**Figure 3:**
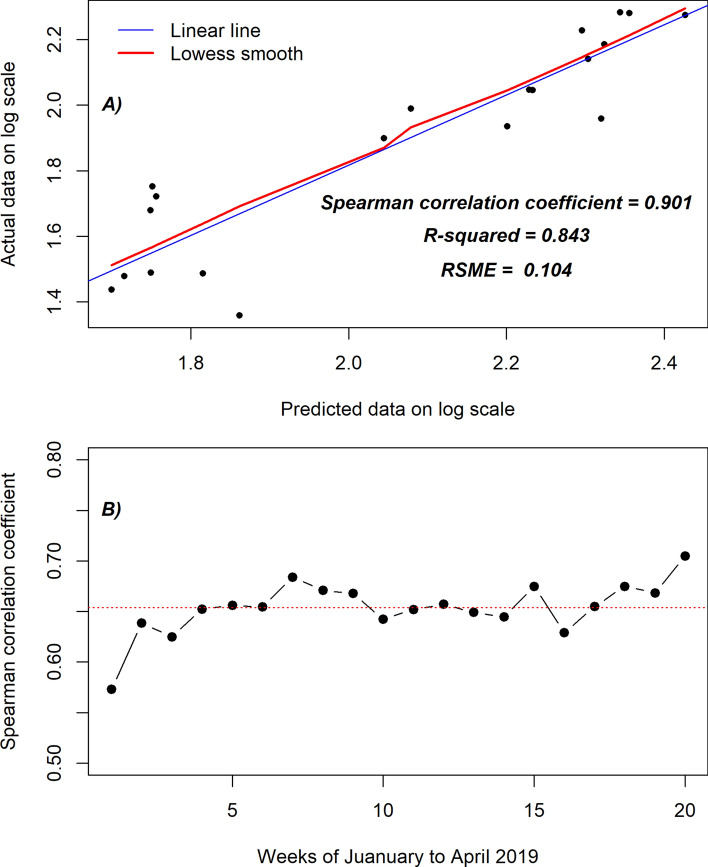
Metric measures for the accuracy of the clinical malaria prediction. **(A)** Linear and smooth relationship between the actual observed data collected during the first quarter (20 weeks) of the year 2019 and predicted values of clinical malaria cases during the same period. **(B)** Spearman correlation coefficient per week between the actual observed data collected during the first quarter (20 weeks) of the year 2019 and predicted values of malaria cases.

The metric that measures the accuracy of the clinical malaria case prediction shows that our proposed model accurately predicted weekly clinical malaria incidence (Fig. 3). This is highlighted by the estimated Spearman correlation coefficient of 0.90, R^2^ of 0.84 and RSME of 0.10.

### Prediction of the weekly clinical malaria incidence

**Figure 4:**
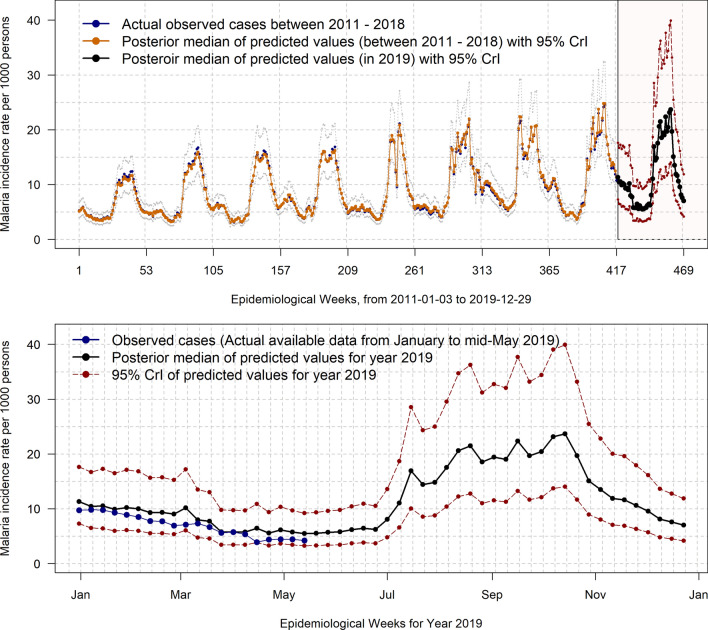
Observed, fitted and predicted values of weekly clinical malaria cases nationwide in 2019 obtained from hierarchical Bayesian spatiotemporal modeling. The bottom image zooms in on the 52 weeks (418–469) of 2019.

National aggregated smoothed values of weekly clinical malaria incidence (both fitted and missing value prediction) estimated from the Bayesian models are presented in Fig. 4. The predicted absolute malaria case count number for the whole nation was estimated at 12,240,063 cases (CrI: 7,283,517–20,570,200) in 2019. The weekly specific absolute malaria case count number for each health district and their uncertainties are provided in the Supplementary Materials (Table S2).

From the predicted values for the year 2019, the national incidence rate was estimated at 605 (95% CrI 360 to 990) per 1000 inhabitants, which represents an average increase of 24.7% (95% CrI 2.4 to 41.6) compared with 2015. However, there was a slight, non-significant increase (0.75%, 95% CrI − 28.5 to 23.0) compared with 2018. The results showed that the weekly specific risk (temporal trend) was constant throughout the year, with increased peaks (twofold) during the rainy seasons (Figure S5).

**Figure 5:**
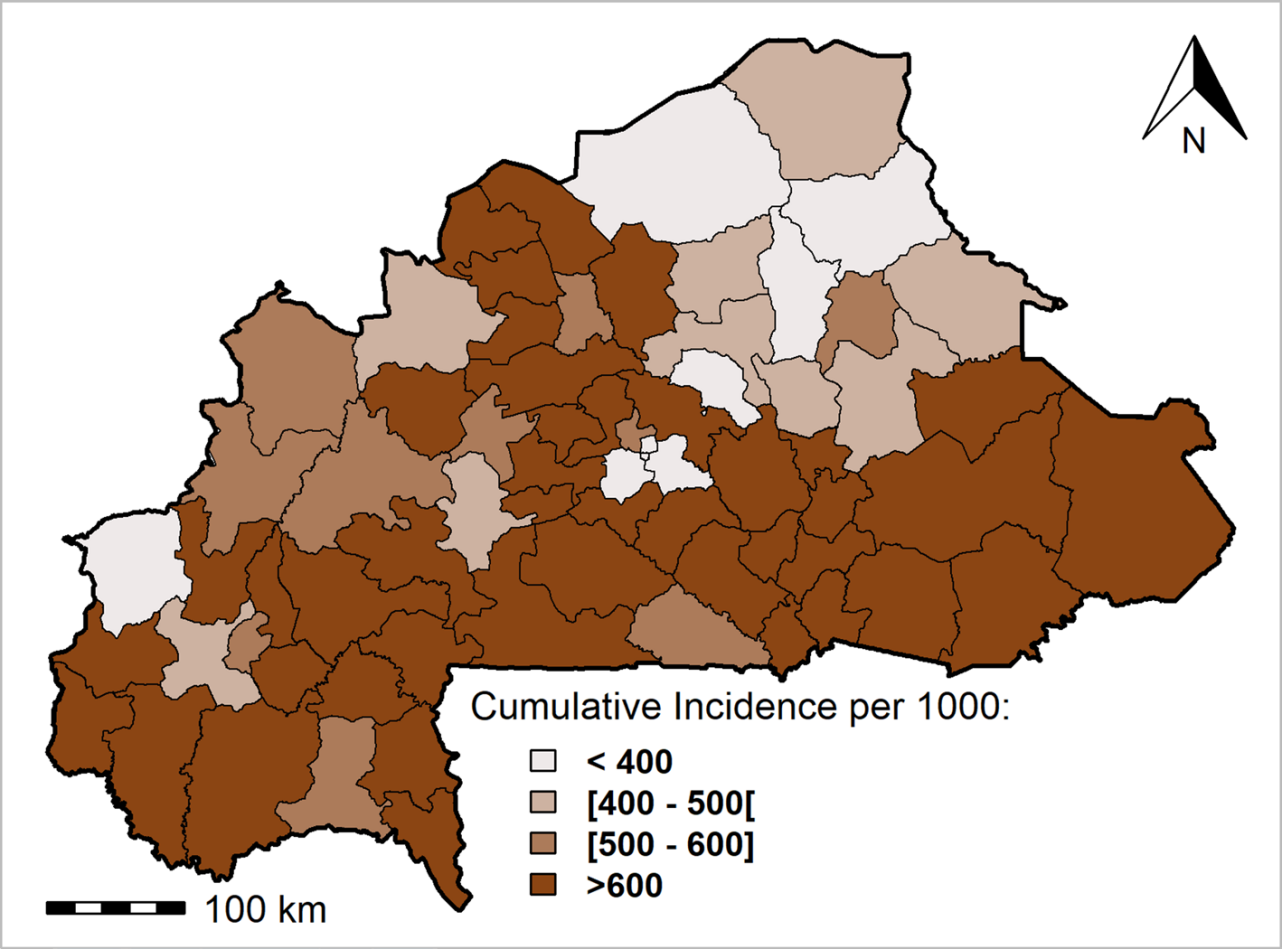
The clinical malaria incidence for each health district of Burkina Faso in 2019. Clinical malaria incidence is the number of cases per 1000 inhabitants. The number of clinical malaria cases for each district of Burkina Faso was predicted using Bayesian spatiotemporal modeling from historical malaria data obtained from the national Official Weekly Telegram. The population data for each district of Burkina Faso were download from worldPop^48^. Maps created by Rouamba T. et al., 2020. The data analysis was carried out at the health district level with no reference to individual-level identification particulars.

Regarding clinical malaria incidence progression, in terms of reduction, the results showed that about 14.3% (10/70) of health districts had reduced their clinical malaria incidence in 2019 compared with 2015. Figure 5 shows clinical malaria incidences for each health district of Burkina Faso in 2019. Nearly 60% (42/70) of health districts exhibited incidence rates of more than 600 cases per 1000 inhabitants. Overall, one-sixth of health districts (12/70) increased by more than 50% of their incidence between 2015 and 2019. These health districts were heterogeneously distributed across the whole country and especially concentrated in the south-west (Dano, Kampti), east (Fada N’Gourma, Gayérie), central west (Sabou, Tenado), central north (Tougouri, Bousouma), Haut-Bassins (N’Dorola) Cascade (Mangodara), Plateau central (Zorgho) and north (Thiou) regions of the country.

## Discussion

Using historical weekly clinical malaria data to address the challenges in routine health data reporting through Bayesian spatiotemporal modeling, we found an increase in clinical malaria incidence nationwide in 2019 compared with previous years. This increase in clinical malaria was heterogenous throughout the country and does not mean that transmission was on the rise in the overall population. Health programs seeking to control malaria, namely the SMC strategy, had a significant association with reduced malaria clinical incidence, whereas free healthcare (resulting in an increase in the rate of health facility attendance) had a significant association with increased malaria clinical incidence. In the present study, it is notable that the interaction between free healthcare and health facility attendance (in other words, the combination of physical and financial access) was associated with a reduction in clinical malaria incidence.

This study is the first attempt to apply a Bayesian spatiotemporal approach aiming to overcome the shortcomings of classical predictive models, which are widely used in the planning and prediction of targets in disease control in the Burkina Faso context^16^. The proposed prediction approach could be applied to other national data in Burkina Faso as well as in other settings to fill the gaps in cases of missing data, with the aim of having a continuous monitoring system while providing guidance for bottleneck strategies (areas with higher underreported rates, field investigations in areas where incidence is stubbornly high). Consequently, the scarce resources in the health system could be redirected/allocated to target the prioritized areas in addition to maintaining and strengthening the currently established health programs. This approach could also be used in the event of natural events or disasters, in which the health system might fail to function effectively (floods, fires) or emergencies (war, outbreaks).

Compared with the actual observed number of clinical malaria cases, our model accurately fitted the incidence of malaria and provided accurate long-range predicted values of weekly malaria distributions for each health district. From the 15th week of 2019, while remaining within the credibility interval, the actual observed values began to deviate towards the lower limit of the credibility interval from the posterior median predicted values. This could be a starting point to indicate the data completeness issue. However, it is important to stress the high uncertainty of these long-term predicted values, even if the reproduction of the median number of clinical malaria cases appears to be similar to the number of reported cases for the first 20 weeks of 2019. This highlights the significant challenges for developing prediction models in endemic settings where clinical malaria incidence depends on several observable and non-observable factors, such as the entomological inoculation rate, mosquito density and extrinsic incubation period of malaria parasites in the mosquito host, population displacements^54,55^ and health interventions. Since our model was adjusted for temperature, distance to a waterbody, humidity and rainfall, several of these factors could be considered minimal because factors related to the cyclic pattern of disease transmission (mosquito development and bites) are affected by weather variables^41,56–59^.

National health statistics, as well as several studies carried out in Burkina Faso, have shown that treatment-seeking practices have constantly been increasing in the last few years, even in the absence of interventions^38,60,61^. The user fee removal, the utilization of health community-based workers for malaria diagnosis and treatment, and the gradual increase in the number of health facilities (from 2278 to 2819 between 2011 and 2019) might accelerate this trend. Our findings also showed that the weekly specific risk (temporal trend) of clinical malaria incidence was constant throughout the year, with peaks during the rainy seasons. However, the overall annual risk increased between mid-2016 and 2017. This annual increase was not present after considering the outpatient attendance rate at health facilities. The increase in the attendance rate enabled health facility staff to increase the diagnosis of cases of malaria^28,29,62^.

In Burkina Faso, health programs, namely SMC, seeking to control malaria had a significant effect on reducing incidence^29^, although this reduction seemed to be masked by the increase in health facility attendance rate. Some health districts did not reduce their clinical malaria incidence in 2019 compared with the year before. This involved the health districts located in the south-west, east, central north, central west and north parts of the country. The scaling up of malaria control interventions in all health district areas from 2019 onwards might have had an impact on malaria incidence in these locations. This implies the need to maintain and strengthen control strategies during all periods of the year and especially focus on the six to eight weeks before the periods of peaks (for SMC)^63^.

Public health officials need reliable and regularly updated data to improve subnational and national health readiness to substantially reduce the burden of malaria^8,9^. The actors who drive the functioning of the health system could use the predicted values to plan for peaks in the use of health services by patients and adapt their functioning accordingly. Similarly, this prediction approach could strengthen the existing system for planning or even anticipating the supply of diagnostic and treatment equipment, especially for areas that suffer from a lack of adequate logistical capacities and inaccessible roads during certain periods of the year^64^.

With the health workers’ strike, individuals with episodes of fever might not have had access to optimal malaria diagnosis, and malaria cases would not have been treated. These individuals would represent an increased reservoir of parasites within communities, which would be expected to lead to an increase in the entomological inoculation rate. In our present study, we assumed that malaria transmission during the health workers strike would be similar to that experienced in previous years because the population still sought care or treatment from other sources, such as the private sector, self-treatment (household treatment) or traditional/herbal medicines. Indeed, a study revealed that about 69% of treatments for fever episodes took place in a patient’s household in the Burkina Faso setting^65^. Similarly, the utilization of plants as remedies is deeply anchored in the social structure of Burkinabe, as evidenced by a study that estimated that 90% of the population relied on traditional remedies for healthcare^66^. Furthermore, according to the WHO, up to 80% of the population living in developing countries relies on traditional/herbal medicines for their primary care needs^67^. Although the utilization of health products originating from traditional/herbal medicines seems to have decreased over the last three decades, there is no doubt that this practice remains an important element in Burkinabe society and a major source of medicines for a large part of the population^68,69^. It was also reported that during the strike period, routine activities in the health facilities operated almost as usual, other than the fact that data on disease surveillance (OWT) and activity reports were not transmitted.

The strengths of our study are that it used weekly data at the health district level for a long period (469 weeks). In addition, our modeling approach, which considers health program data, the seasonality of malaria transmission, spatial and temporal trends as well as environmental impact or unmeasured factors, provided reproducible estimates of clinical malaria data. In our prediction approach, control factors in the model were included for several purposes. The effectiveness of SMC on malaria risk reduction has already been demonstrated in other randomized or quasi-experimental studies in Burkina Faso^42,43^ as well as in other sub-Saharan African countries^63^. Previous studies also showed that the implementation of the free healthcare policy was significantly associated with a reduction in clinical malaria incidence. However, in Burkina Faso, the free healthcare policy also resulted in both a substantial increase in the health facility attendance rate and the availability of malaria diagnostic tools^29^. This has enabled health facilities to diagnose clinical malaria cases more comprehensively. The model was adjusted for the health facility attendance rate as well as the proportion of the most vulnerable population (young children and pregnant women). This allowed for a smooth adjustment of the model and consideration of these covariates and weather factors when predicting the number of cases.

Although our study provides useful insights, a few limitations exist. First, the current malaria data reported in the framework of weekly surveillance are a combination of confirmed cases and clinical cases. Since malaria data for this study were from the OWT (confirmed and clinically diagnosed cases), the incidence estimates could be improved if the total number of RDTs performed are controlled for because the increased use of RDTs would lead to a reduction in incidence, as some people previously assumed to have malaria would test negative. However, these data are not reported in the OWT; those available in the DHIS2 (monthly data) did not cover the entire period of our study. Second, the denominators (population at risk) in each health district were not from census data and were obtained from WorldPop, and this might have led to over- or under-estimation of the number of populations at risk. Third, the relative complexity of this kind of analysis requires some level of statistical skills to interpret the Bayesian model outputs, and this expertise is not always present in NMCP teams. Fourth, to improve prediction accuracy, it was important to account for the internal migration and movement of populations in neighboring countries, as well as entomological data in the model, but such data were not yet available for Burkina Faso at the health district level. Finally, the CAR models applied to the routine data in our study are known to be subject to bias due to ecological error^70^. However, the modeling approach that we employed enabled us to account for unmeasured confounders and the uncertainty of the estimates, as well as the potential bias due to the different data sources, allowing us to obtain robust estimates.

## Conclusion

Our study provided an approach that could be deployed to respond to crisis situations that would affect routine data collection procedures. Our study shows that modeling of routinely collected surveillance health data can be beneficial in the broad prediction of specifically-timed disease both at the national and subnational levels. The predicted values for each health district, as well as the pinpointed high-intensity areas and periods for malaria, should contribute to strengthening the resilience and sustainability of the healthcare system and inform future control planning and research. The provided analytical approach on malaria data could be not only an alternative to address the challenge of malaria routine surveillance data completeness but also useful tools for anticipating interventions and forecasting specific needs in malaria control resources. The findings reveal an increase in the clinical malaria incidence rate in 2019 compared with the year before, and only one-eighth of health districts had reduced their clinical malaria incidence in 2019 compared with 2015. Furthermore, our findings will contribute to reconsidering the goals and targets of the NMCP during the updating of the "National technical strategy for malaria 2021–2025". Finally, but not the last, the proposed modeling approach could also be adapted and used to address the challenge of routine health data completeness of other health indicators.

## Supplementary information

Supplementary Figure S1.

Supplementary Figure S2.

Supplementary Figure S3.

Supplementary Figure S4.

Supplementary Figure S5.

Supplementary Table S1.

Supplementary Table S2.

## Data Availability

The dataset regarding malaria cases was provided by the National Malaria Control Program. The datasets generated during and/or analyzed during the current study are available from the corresponding author upon reasonable request.
