# Supplementary material for: Addressing challenges in routine health data reporting in Burkina Faso through Bayesian spatiotemporal prediction of weekly clinical malaria incidence
Source: Sci Rep. 2020 Oct 6;10:16568. doi: 10.1038/s41598-020-73601-3 (PMC7538437; doi:10.1038/s41598-020-73601-3)
Supplement: Supplementary file 2 — Supplementary Figure S2. [file 41598_2020_73601_MOESM2_ESM.pdf]

Addressing Challenges in Routine Health Data Reporting in Burkina Faso through Bayesian Spatiotemporal Prediction of Weekly Clinical Malaria Incidence

Toussaint Rouamba[1,2], **Sekou Samadoulougou**[3,4] **and Fati Kirakoya–Samadoulougou**[2]

1 Clinical Research Unit of Nanoro, Institute for Research in Health Sciences, National Center for Scientific and Technological Research, 42, Avenue Kumda-Yoore, BP 218 Ouagadougou CMS 11, Ouagadougou, Burkina Faso

2 Center for research in epidemiology, Biostatistics and Clinical Research, School of Public Health, University libre de Bruxelles (ULB), Route de Lennik, 808 B-1070 Bruxelles. Brussels, Belgium

3 Evaluation Platform on Obesity Prevention, Quebec Heart and Lung Institute, Quebec, G1V 4G5, Canada

4 Centre for Research on Planning and Development (CRAD), Laval University, Quebec, G1V 0A6, Canada

*Correspondence to rouambatoussaint@gmail.com

Supplementary material 2

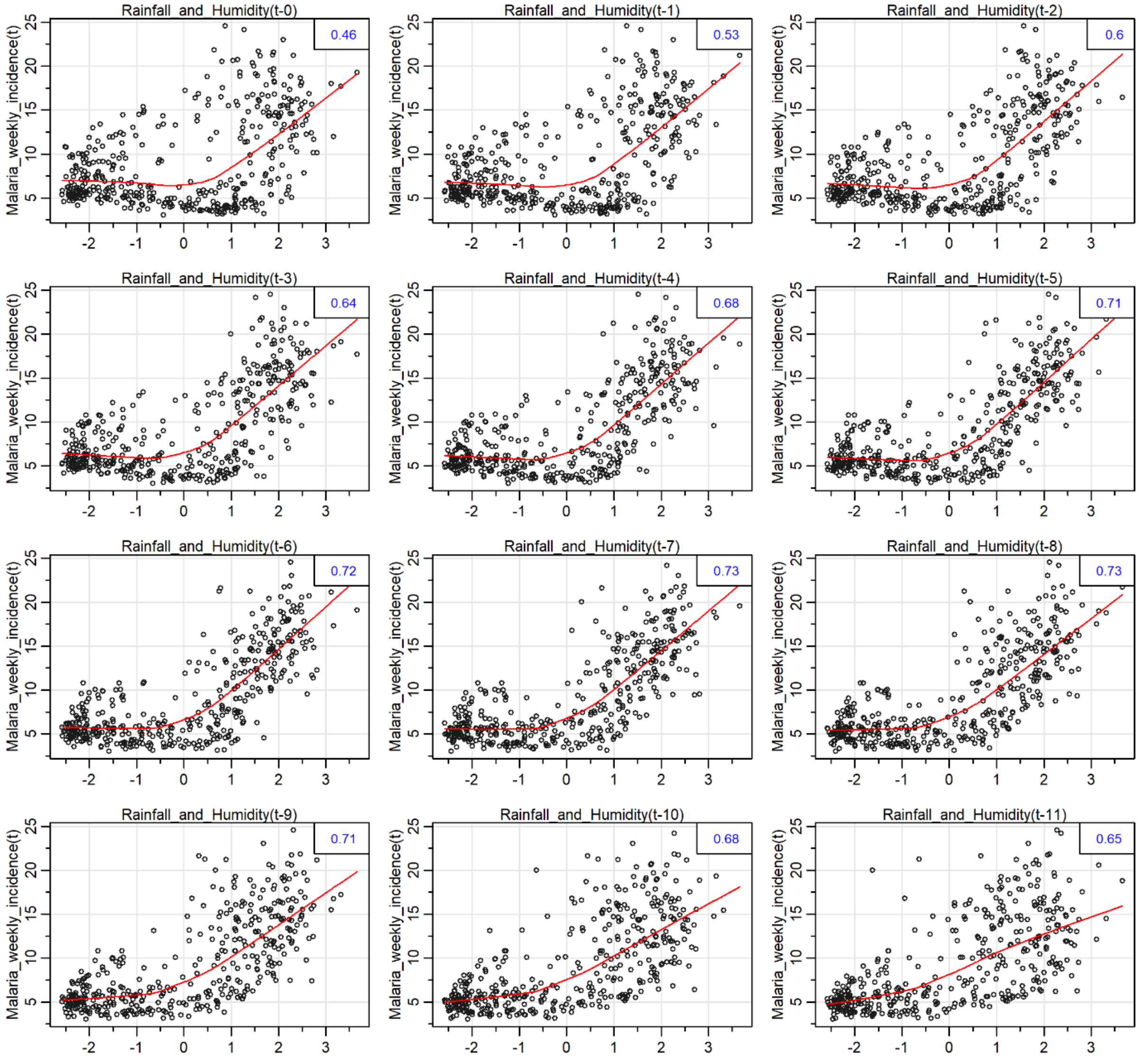

Figure S2. Relationship (cross-correlation) between malaria incidence rate and first component (resumes Rainfalls and relative humidity) from principal component analysis. The red curves represent the smooth relationships between incidence rates according to the first component values considering the lag-times. The number on the top right within each box represent the correlation coefficient
