# Supplementary material for: Addressing challenges in routine health data reporting in Burkina Faso through Bayesian spatiotemporal prediction of weekly clinical malaria incidence
Source: Sci Rep. 2020 Oct 6;10:16568. doi: 10.1038/s41598-020-73601-3 (PMC7538437; doi:10.1038/s41598-020-73601-3)
Supplement: Supplementary file 3 — Supplementary Figure S3. [file 41598_2020_73601_MOESM3_ESM.pdf]

Addressing Challenges in Routine Health Data Reporting in Burkina Faso through Bayesian Spatiotemporal Prediction of Weekly Clinical Malaria Incidence

Toussaint Rouamba[1,2], Sekou Samadoulougou[3,4] and Fati Kirakoya–Samadoulougou[2]

1 Clinical Research Unit of Nanoro, Institute for Research in Health Sciences, National Center for Scientific and Technological Research, 42, Avenue Kumda-Yoore, BP 218 Ouagadougou CMS 11, Ouagadougou, Burkina Faso
2 Center for research in epidemiology, Biostatistics and Clinical Research, School of Public Health, University libre de Bruxelles (ULB), Route de Lennik, 808 B-1070 Bruxelles. Brussels, Belgium
3 Evaluation Platform on Obesity Prevention, Quebec Heart and Lung Institute, Quebec, G1V 4G5, Canada
4 Centre for Research on Planning and Development (CRAD), Laval University, Quebec, G1V 0A6, Canada
*Correspondence to rouambatoussaint@gmail.com

Supplementary material 3

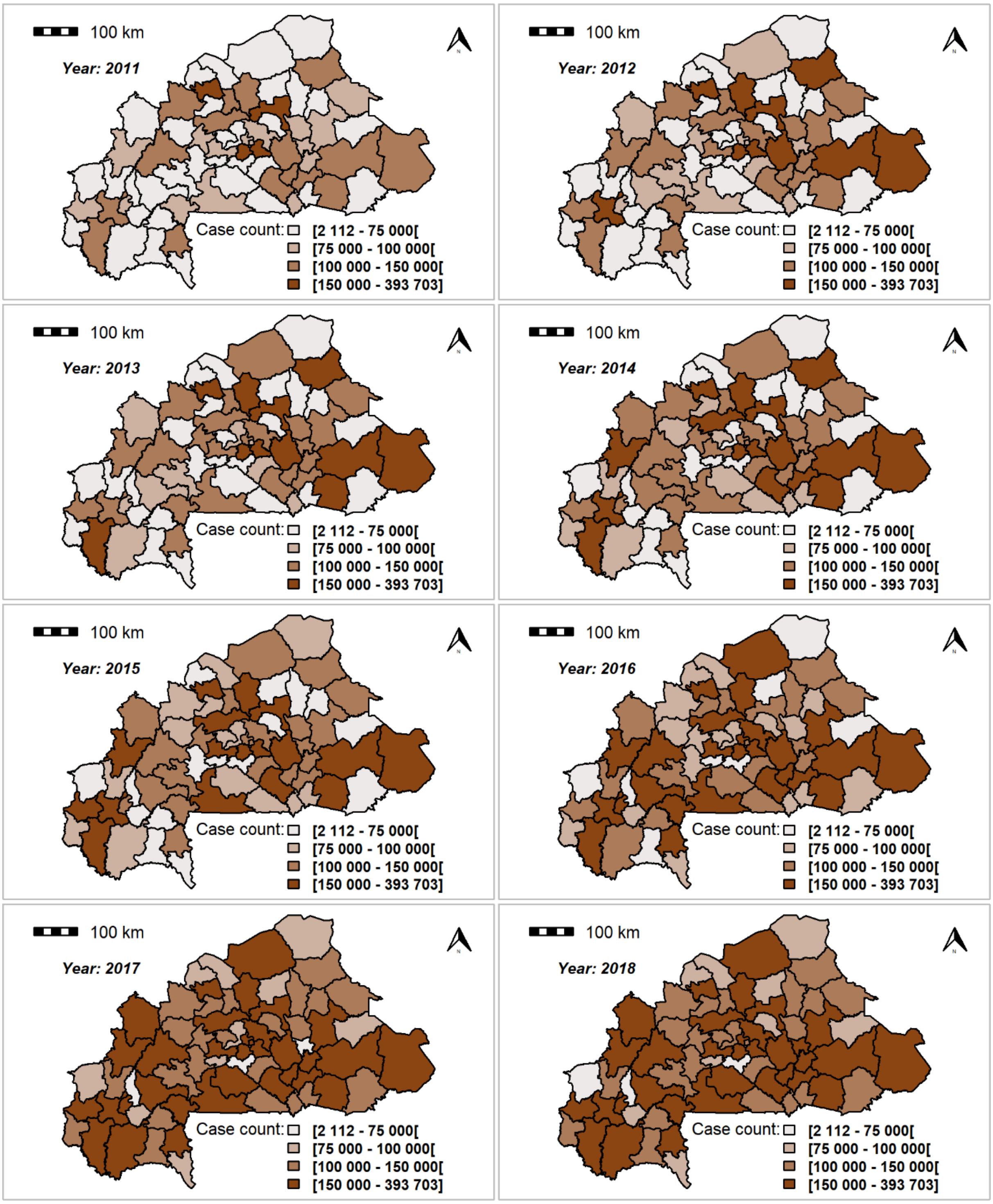

Figure S3. The malaria absolute cases cunt number for each health-district of Burkina Faso from 2011 to 2018.
The number of malaria cases for each district of Burkina Faso from 2011 to 2018 were collected from the national Official Weekly Telegram.
The population data for each district of Burkina Faso were download from worldpop[42]
Maps created by Rouamba T. et al 2020
