# Supplementary material for: Addressing challenges in routine health data reporting in Burkina Faso through Bayesian spatiotemporal prediction of weekly clinical malaria incidence
Source: Sci Rep. 2020 Oct 6;10:16568. doi: 10.1038/s41598-020-73601-3 (PMC7538437; doi:10.1038/s41598-020-73601-3)
Supplement: Supplementary file 5 — Supplementary Figure S5. [file 41598_2020_73601_MOESM5_ESM.pdf]

Addressing Challenges in Routine Health Data Reporting in Burkina Faso through Bayesian Spatiotemporal Prediction of Weekly Clinical Malaria Incidence

Toussaint Rouamba[1,2], Sekou Samadoulougou[3,4] and Fati Kirakoya–Samadoulougou[2]

1 Clinical Research Unit of Nanoro, Institute for Research in Health Sciences, National Center for Scientific and Technological Research, 42, Avenue Kumda-Yoore, BP 218 Ouagadougou CMS 11, Ouagadougou, Burkina Faso
2 Center for research in epidemiology, Biostatistics and Clinical Research, School of Public Health, University libre de Bruxelles (ULB), Route de Lennik, 808 B-1070 Bruxelles. Brussels, Belgium
3 Evaluation Platform on Obesity Prevention, Quebec Heart and Lung Institute, Quebec, G1V 4G5, Canada
4 Centre for Research on Planning and Development (CRAD), Laval University, Quebec, G1V 0A6, Canada

*Correspondence to rouambatoussaint@gmail.com

Supplementary material 5

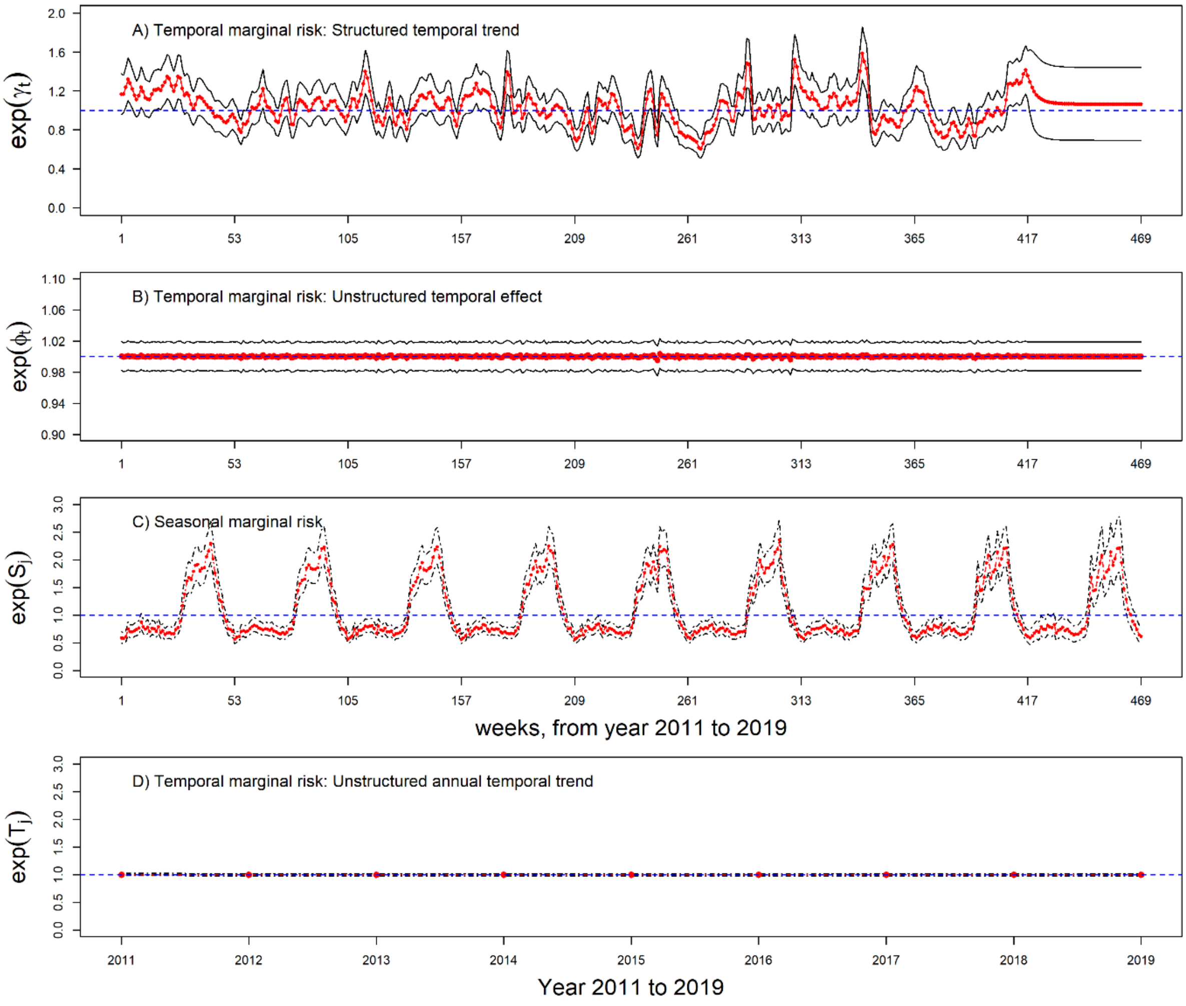

Figure S5. Overall temporal marginal risk from full model for weekly malaria incidence in Burkina Faso with 95% CrI: A) Weekly temporally structured trend $exp(\gamma_t)$, B) Weekly temporally unstructured trend $exp(\phi_t)$, C) Seasonal effect $exp(S_j)$ and), D) Annual temporally unstructured trend $exp(T_j)$
