# Supplementary material for: Addressing challenges in routine health data reporting in Burkina Faso through Bayesian spatiotemporal prediction of weekly clinical malaria incidence
Source: Sci Rep. 2020 Oct 6;10:16568. doi: 10.1038/s41598-020-73601-3 (PMC7538437; doi:10.1038/s41598-020-73601-3)
Supplement: Supplementary file 6 — Supplementary Table S1. [file 41598_2020_73601_MOESM6_ESM.pdf]

Addressing Challenges in Routine Health Data Reporting in Burkina Faso through Bayesian Spatiotemporal Prediction of Weekly Clinical Malaria Incidence

Toussaint Rouamba[1,2], Sekou Samadoulougou[3,4] and Fati Kirakoya–Samadoulougou[2]

1 Clinical Research Unit of Nanoro, Institute for Research in Health Sciences, National Center for Scientific and Technological Research, 42, Avenue Kumda-Yoore, BP 218 Ouagadougou CMS 11, Ouagadougou, Burkina Faso
2 Center for research in epidemiology, Biostatistics and Clinical Research, School of Public Health, University libre de Bruxelles (ULB), Route de Lennik, 808 B-1070 Bruxelles. Brussels, Belgium
3 Evaluation Platform on Obesity Prevention, Quebec Heart and Lung Institute, Quebec, G1V 4G5, Canada
4 Centre for Research on Planning and Development (CRAD), Laval University, Quebec, G1V 0A6, Canada

*Correspondence to rouambatoussaint@gmail.com

Supplementary material 6

Tale S1. Deviance Information criteria values according to the model

Spatiotemporal model	Deviance Information criteria values
Null Model	466 109
Null Model+ $\beta_w X_{kt}$	451 305
Null Model + $\beta_w X_{kt} + \beta_{HP} X_{kj}$	450 906
Null Model +$\beta_w X_{kt} + \beta_{HP} X_{kj} + \beta_{env} X_k$	450 404
Null Model +$\beta_w X_{kt} + \beta_{HP} X^*{}_{kj} + \beta_{env} X_k$	449 843

Null Model: $log(\mu_{kt}) = \beta_0 + log(O_{kt}) + \psi_k + \omega_t + \delta_{kt} + T_j + S_j$

X_{kt} : rainfall, temperature, relative humidity

X_{kj} : SMC duration, free of charge of health care, poverty index, children under five year and pregnant women, health-facility attendance rate

$X^*{}_{kj}$: included interaction effect of charge of health care and facility attendance rate

X_k : average distance to land water
